# Characterising processes and outcomes of tailoring implementation strategies in healthcare: a protocol for a scoping review

**DOI:** 10.12688/hrbopenres.13507.2

**Published:** 2022-09-20

**Authors:** Fiona Riordan, Claire Kerins, Nickola Pallin, Bianca Albers, Lauren Clack, Eimear Morrissey, Geoffrey M. Curran, Cara C. Lewis, Byron J. Powell, Justin Presseau, Luke Wolfenden, Sheena M. McHugh

**Affiliations:** 1 School of Public Health, University College Cork, Cork, Cork, T12 XF62, Ireland; 2 Institute for Implementation Science in Health Care, University of Zurich, Zurich, Switzerland; 3 School of Medicine, School of Psychology, National University of Ireland, Galway, Ireland; 4 Department of Psychiatry, University of Arkansas for Medical Sciences, Little Rock, Arkansas, USA; 5 Kaiser Permanente Washington Health Research Institute, Seattle, Washington, USA; 6 Brown School, Washington University in St. Louis, St. Louis, Missouri, USA; 7 Clinical Epidemiology, Ottawa Hospital Research Institute, Ottawa, Canada; 8 School of Medicine and Public Health, Faculty of Health and Medicine, University of Newcastle,, New South Wales, Australia

**Keywords:** implementation science, implementation strategies, tailoring, scoping review, protocol

## Abstract

**Background:** Tailoring strategies to target the salient barriers to and enablers of implementation is considered a critical step in supporting successful delivery of evidence based interventions in healthcare. Theory, evidence, and stakeholder engagement are considered key ingredients in the process however, these ingredients can be combined in different ways. There is no consensus on the definition of tailoring or on a single method for tailoring strategies to optimize impact, ensure transparency, and facilitate replication.

**Aim:** The purpose of this scoping review is to describe how tailoring has been undertaken within healthcare to answer questions about how it has been conceptualised, described, and conducted in practice, and to identify research gaps.

**Methods:** The review will be conducted in accordance with best practice guidelines and the Preferred Reporting Items for Systematic Reviews and Meta-analysis extension for scoping reviews (PRISMA-ScR) will be used to guide the reporting. Searches will be conducted of MEDLINE, Embase, Web of Science, Scopus, from 2005 to present. Reference lists of included articles will be searched. Grey literature will be searched on Google Scholar. Screening and data extraction will be conducted by two or more members of the research team, with any discrepancies resolved by consensus discussion with a third reviewer. Initial analysis will be quantitative involving a descriptive numerical summary of the characteristics of the studies and the tailoring process. Qualitative content analysis aligned to the research questions will also be conducted, and data managed using NVivo where applicable. This scoping review is pre-registered with the Open Science Framework.

**Conclusions:** The findings will serve as a resource for implementation researchers and practitioners to guide future research in this field and facilitate systematic, transparent, and replicable development of tailored implementation strategies.

## Introduction

Adoption and subsequent health impact of evidence-based interventions (EBIs) is facilitated by the careful use of implementation strategies which are “methods or techniques used to enhance the adoption, implementation, and sustainment of a clinical program or practice” ^1^. It is recommended that such strategies are purposefully selected and designed to better address the specific needs within a given implementation context, be these at the level of the patient, provider, organisation, and/or system. Numerous possible implementation strategies have been proposed. For example, 73 discrete strategies were compiled by the Expert Recommendations on Implementing Change (ERIC) study ^2^. However, a process is necessary to choose between them and operationalise them for a given setting. Tailoring is one process which involves selecting and/or modifying strategies to address contextual factors that potentially influence implementation ^3^. It is considered a critical step in supporting successful delivery of EBIs in healthcare ^4^. 

However, there are varying definitions and conceptualisations of tailoring and it has been suggested that tailoring is a “poorly developed concept” within implementation science ^5^. The term ‘tailoring’ has most commonly ^4^ been used to describe a proactive approach to customize the method of implementation (i.e. the strategy), however, the term has also been used to describe the process of modifying or personalising strategies to fit with different population subgroups ^6– 8^, or adapting strategies during delivery ^9^. 

Tailoring as a process is also somewhat ambiguous as the timing, level, scope and methods of application varies and there are no generally accepted guiding principles for tailoring to ensure transparency and facilitate replication. There are two levels to how the tailoring process is operationalised: (1) the individual steps to take, by whom, when and how often (timing), and (2) the overall approach, or way in which those steps are taken, including the research methods and data collection techniques used. The steps included in the tailoring process vary, with studies including some or all of the following: (a) the identification of (barriers and enablers) implementation determinants, (b) prioritisation of determinants, and (c) selection of strategies by matching to determinants ^6– 8, 10^ and modifying or adjusting these strategies ^3^. It is also unclear whether application and testing of strategies forms part of the tailoring process ^3, 9, 11^. It is generally recommended that there should be some identification of implementation determinants, but in isolation that is insufficient; there should be a corresponding process to select strategies to address those factors ^4^. In terms of the timing, it is unclear whether tailoring is an initial design process that should take place before deployment of an implementation strategy, as specified by Baker *et al*. ^4^, or whether it can be an iterative process that continues during strategy deployment as implementation challenges arise ^12, 13^. The Template for Intervention Description and Replication (TIDieR) checklist distinguishes between tailoring (personalisation and planned adaptations) and modification (unplanned modifications over the course of the study) ^14^. This contrasts with studies that have used the Knowledge-To-Action (KTA) ^15^ translation cycle to engage in iterative tailoring to modify strategies, or the Model of Implementation which has been used to undertake more than one round of tailoring, informing further changes to the original strategy in response to emerging barriers ^16, 17^. The Framework for Reporting Adaptations and Modifications to Evidence-based Implementation Strategies (FRAME-IS) refers to the modification of strategies after deployment as ongoing tailoring ^18^, while efforts to identify and develop practical tools for tracking strategies, distinguish between tailoring as prospective design of strategies, and adaptations as ‘deliberate modifications’ to the treatment or how it is delivered ^19^.

The overall approach to tailoring, that is, how tailoring has been applied, the way in which the steps have been delineated and the research methods (e.g. intervention mapping) and data collection techniques used (e.g., surveys, focus groups), has not been consistent or well described ^3^. A Cochrane review of the effectiveness of tailored implementation strategies, found tailoring was undertaken in different ways, the methods used varied substantially, as did the rationale for their selection, they lacked detail, and trials often did not outline the underpinning rationale for tailoring ^4^. Powell *et al.* ^3^, described four approaches that have been used for prospective tailoring ranging from multiphase approaches (intervention mapping) to specific analytic tools (concept mapping, conjoint analysis and group model building). These approaches, which often involve several steps and types of data, were selected on the basis that they had been used to develop interventions in contexts outside of implementation and behavioural science, where extensive literature is available to guide their use, and approaches are not proprietary. While existing approaches to tailoring, like those outlined by Powell *et al.*, share common elements (theory, evidence, and stakeholder engagement) and steps (determinant identification, determinant prioritisation, selection of strategies to address determinants, and application of the selected strategy), there is no consensus on how to combine these elements of tailoring and what each element should involve ^3, 20^. Work has been conducted as part of the Tailored Implementation in Chronic Diseases (TICD) study to investigate the methods used to identify determinants, concluding that brainstorming is a feasible approach which can yield a high number of plausibly important determinants ^21^. However, there have been calls for greater clarity on how determinants are prioritised, and how strategies are selected ^22^.

### Purpose of conducting the scoping review

Despite varying definitions and an apparent lack of clarity about how to best conduct it in practice, tailoring is commonly recommended to support the successful delivery of EBIs. Research to date has been systematically reviewed to establish the effectiveness of tailored strategies, the outcome of the tailoring process. The literature has not been reviewed to map the description and application of this process, to establish a clear picture of how tailoring has been conceptualised and conducted in practice, including the contexts in which tailoring has been used, when it has been used, and who has been involved in the process ^3^. Our aim is to address this gap by conducting a scoping review to: 1) explore how tailoring has been defined and conceptualised in the healthcare literature; 2) examine how tailoring has been operationalised within the healthcare context (timing, level and scope); 3) determine how tailoring has been evaluated in the context of healthcare; and 4) identify knowledge gaps and future research priorities. As the aim of this review is to explore how tailoring has been defined and conceptualised, we adopt a broad definition of tailoring as a proactive process to develop the method of implementation, and/or modifications of an implementation strategy to fit with different population subgroups or emerging barriers. Scoping reviews are defined by Colquhoun *et al.,* as “a form of knowledge synthesis that addresses an exploratory research question aimed at mapping key concepts, types of evidence, and gaps in research related to a defined area or field by systematically searching, selecting, and synthesizing existing knowledge” ^23^ providing essentially a ‘map of the evidence’ ^24^. By clarifying how tailoring is conceptualised and summarising approaches to tailoring, this review will serve as a resource for the implementation research and practice community who are designing implementation strategies to address a particular implementation gap in healthcare. It will identify research priorities on tailoring and may serve as a starting point from which to develop and agree a consolidated definition of tailoring.

## Protocol

### Design

The review will be conducted in line with the framework and principles of Arksey and O’Malley ^25^ and the refinements proposed by Levac *et al*. ^24^, We will also draw on updated guidance developed by the Joanna Briggs Institute (JBI) institute ^26^. The Preferred Reporting Items for Systematic Reviews and Meta-Analyses extension for Scoping Reviews (PRISMA- ScR) guidelines will be followed. There will be six stages in the review process: 1) Identifying the research questions; 2) Identifying relevant studies; 3) Study selection; 4) Data extraction/charting the data; 5) Collating, summarising, and reporting results, and 6) Stakeholder consultation.

***Stage 1: identifying the research questions***

 A preliminary search of relevant literature was undertaken in one database (searching MEDLINE) using “tailoring” and “implementation” and “strategy” or “intervention” to generate an initial understanding of how tailoring has been operationalised. These findings (e.g., existing editorials and reviews of tailoring, examples of tailoring approaches) were used to inform the background for the review, refine the scope of the review, generate eligibility criteria, and to develop the search strategy.

The following objectives and research questions were identified through this iterative process:

**Objective 1: Conceptualisation**

1. How has tailoring of implementation strategies (i.e., methods or techniques used to enhance the adoption, implementation, and sustainment of a clinical program or practice), been defined and conceptualised in healthcare contexts?
2. Is there a rationale for tailoring and if so, what is the rationale?
3. Is tailoring informed by a theory which explains why it should work?

**Objective 2: Operationalisation**

1. What steps comprise the tailoring process?
2. Which research methods (e.g., intervention mapping, conjoint analysis) and data collection techniques have been used?
3. Which theoretical frameworks have been drawn on and at which steps(s)?
4. When was tailoring carried out (stage of implementation)?
5. Where was tailoring been carried out (in which healthcare settings)
6. Who was involved, when (what step(s)), for what purpose, and how?
7. How was the prioritisation of determinants and selection of strategies conducted?
  a. What methods have been used and what is the rationale?
  b. What are the inputs during strategy selection – evidence, stakeholders, theory?
  c. What is the balance and timing of each input?
  d. What criteria have been used in prioritisation?
  e. Who selected these criteria?

**Objective 3: Evaluation**

1. How has the tailoring process been evaluated in healthcare?
2. Which study designs, including comparison conditions, have been used to evaluate tailoring?
3. What proximal outcomes of tailoring “success” have been used?

Through seeking to answer the above questions we will determine gaps in the literature (Objective 4) which need to be addressed as part of future research on tailoring and inform a research agenda focused on tailored approaches to implementation (Objective 5).

***Stage 2: identifying relevant studies***

**Eligibility criteria**

The ‘‘PCC’’ mnemonic (population, concept, and context) is recommended by the Joanna Briggs Institute to construct clear inclusion criteria for scoping reviews and identify the focus and context of the review ^26, 27^.

*Population*

All populations involved in health care research are relevant. This could include healthcare professionals, implementation researchers, and other people who plan, provide and/or use health services

*Concept*

In defining the concept for this review, we made several decisions to make the process more feasible ( Table 1). Studies which use a tailoring process to develop implementation strategies in healthcare will be included. As one of our objectives is to examine how tailoring has been conceptualised, we will adopt a broad definition of the process for the purpose of identifying relevant studies. To address objective 1 (conceptualisation), we will consider primary studies *and* commentaries or editorials which discuss how tailoring should be done and/or why. To address objectives 2 (operationalisation) and 3 (application), we will only consider primary studies which use tailoring as a prospective process to develop the method of implementation, or modifications of a strategy to fit with different population subgroups (e.g., socio-demographic characteristics; age, gender, culture, socio-economic status) or individual sites. For primary studies to be considered tailoring, approaches must at least involve the selection of strategies. While there is variation in the scope of tailoring (i.e., whether determinant identification and/or prioritisation, and strategy selection, and/or application and testing are included), strategy selection is a core aspect of tailoring. Authors must explicitly describe the process as tailoring or the strategy as tailored. Studies will also be included if they refer to the intervention/strategy development process as ‘implementation mapping’. This term was adapted from ‘intervention mapping’ and first used by Fernandez *et al.* ^28^ to specifically describe an approach to tailor implementation strategies. In line with guidance specified by Levac *et al.* ^24^, who advise the need to balance breadth and comprehensiveness with feasibility, we place these bounds on the concept to manage the scope of the review. For example, there is risk of unearthing a large volume of literature focused only on determinant identification without subsequent processes to tailor strategies.

**Table 1.** Eligibility criteria for the review.

Criterion	Inclusion	Exclusion	Justification
**Population**	All populations involved in health care research are relevant. This could include healthcare professionals, implementation researchers, and other people who plan, provide and/or use health services.	Other populations.	Focus of the research programme is healthcare interventions. Extending beyond healthcare would be unmanageable with review resources.
**Concept**	Commentaries or editorials/opinion pieces which discuss how tailoring should be done and/or why (objective 1) Primary studies that use a tailoring process to prospectively select implementation strategies prior to deployment in healthcare or tailoring of a strategy to fit with different population subgroups (objectives 1-3)	Studies adapting implementation strategies to emergent barriers after deployment.	The aim of the review is to inform priorities for a research programme on prospective tailoring to develop strategies to support the adoption and implementation of healthcare interventions.
**Context**	Studies which use a tailoring process to develop strategies (methods or techniques used to enhance the adoption, implementation, and sustainment of a clinical program or practice), for application in healthcare settings *	Studies using tailoring outside of the healthcare context. For example educational settings.	Focus of the research programme is healthcare interventions. Extending the review beyond healthcare would be unmanageable with the resources available for the review.
**Language**	English	Non-English	Reviewers are only English speaking and do not have the resources to translate articles to other languages.
**Time** ** period**	Year 2005 – 2022	Before 2005	Given the breadth of the search, and to manage resources, the decision was made to focus on over 17 years of literature. The flagship journal in the field, Implementation Science started in 2006, signaling the formal establishment of the field in 2006 and subsequently, dedicated journals, including Implementation Science Communications (2020), Implementation Research and Practice (2020), and Global Implementation Research and Applications (2021) have been established.
**Types of** ** articles**	1. Peer reviewed journal articles of primary studies 2. Unpublished (grey literature) including theses/dissertations, and book chapters, toolkits, guidance, reports. 3. Study protocols 4. Editorial/opinion pieces will be used as source of additional articles and to address objective 1	• Conference abstracts • Non-peer reviewed sources (i.e., presentations, websites, blogs, studies in progress)	Aim to capture more than peer reviewed literature to fully understand how tailoring has been conducted in practice.
**Geographic**	Any location	None	Tailoring implementation strategies has application globally.

*state-funded and private organisations providing services in the following areas: disability, older persons, nursing homes, acute and non-acute hospitals, community hospitals, mental health, social inclusion, palliative care, chronic illness, primary care (GP, dental, pharmacies, physiotherapy clinics), health and wellbeing, hospice, rehabilitation, home care, paramedics, and community services (e.g. youth, substance abuse, suicide prevention, community development)” ^30^.

If studies are identified which operationalise tailoring differently to our broad definition, this will also be documented. For example, studies describing tailoring conducted after deployment of a strategy, adapting delivery to target emerging barriers will be excluded. Studies which describe an approach to tailor an EBI as distinct from a strategy to support implementation of an EBI will be excluded. The difference between the two can be ambiguous ^29^. Further confusion is caused by labels; ‘implementation intervention’ is sometimes used interchangeably with ‘implementation strategy’. We will commit to the definition of strategy cited above: “methods or techniques used to enhance the adoption, implementation, and sustainment of a clinical program or practice” ^1^. For this definition to apply there must be a clearly identifiable intervention (programme, practice, guideline, policy or innovation) being implemented. The focus will not be on the nature or type of intervention being implemented, or whether it should be implemented, rather the focus will be on the tailored strategy supporting implementation. Where the delineation between intervention and strategy is unclear, we will refer to the ERIC taxonomy to identify implementation strategies. At the full text screening stage, if we cannot clearly delineate the intervention, we will contact the study authors for more information. If the author cannot provide more clarity, we will include the study but report the ambiguity.

Studies will only be included if they (a) describe the tailoring approach in some detail, that is, the authors describe at least one aspect of the tailoring approach (i.e., when it was conducted, who was involved, format/method used, steps involved, inputs) and (b) at least involve the selection of strategies.

**Context**

Studies which use a tailoring process to develop strategies (methods or techniques used to enhance the adoption, implementation, and sustainment of a clinical program or practice) for application in healthcare settings will be included. Healthcare settings is defined as “state-funded and private organisations providing services in the following areas: disability, older persons, nursing homes, acute and non-acute hospitals, community hospitals, mental health, social inclusion, palliative care, chronic illness, primary care (GP, dental, pharmacies, physiotherapy clinics), health and wellbeing, hospice, rehabilitation, home care, paramedics, and community services (e.g. youth, substance abuse, suicide prevention, community development)” ^30^. 

Both qualitative and quantitative primary research studies will be eligible. Similarly, commentaries/editorials/opinion pieces will be used as a source of studies and to address objective 1 on conceptualisation. Unpublished (grey literature) will be included e.g., toolkits, guidance, reports. Conference abstracts will be excluded. Only reports and articles published in the English language and between the years 2005 and 2022 will be included. Table 1 outlines the eligibility criteria. A preliminary electronic search for key terms (“tailor*” and terms for strategy or intervention and implementation and healthcare) in title and abstracts along with relevant subject headings in MEDLINE ^14^ indicated that in the last 20 years, there has been an upward trend in articles referring to tailoring, with 1722 articles published 2012–2021 compared to 443 articles in the 10 years (2002–2011) previous. In total, there were 152 articles published 1972–2005 and 2148 from 2006 to 2022. Hence our decision regarding search date limitations.

**Search strategy**

An information scientist (university librarian) will aid the authors to design and refine the search strategy. The search strategy will follow a three step strategy in line with JBI guidance ^31^. First, MEDLINE and Embase will be searched. Text words and index terms contained in the title and abstract of retrieved papers will be compiled to inform subsequent more comprehensive searches. This list will be developed further by considering appropriate thesaurus terms and synonyms. A number of exemplar studies ^9, 21, 32– 36^ will be used to check the search strategy.

Second, a search using all identified keywords (e.g., ‘tailor’ ‘tailoring’) and index (MeSH) terms will be undertaken across four databases, MEDLINE, Embase, Web of Science Core Collection, and Scopus ^37^. Dedicated searches for a specific tailoring approach, ‘implementation mapping’, will be completed for each database. This specific approach was chosen as it is unique to tailoring implementation strategies. Other methods (intervention mapping concept mapping, conjoint analysis, and group model building) while suggested as possible tailoring approaches, can also be used in other fields and for other purposes. This lack of specificity was confirmed during preliminary searches conducted when developing the search. Complete search strategies for each database will be reported as appendices. Third, the reference lists of identified articles and reports will be searched for additional sources and the authors of primary articles or reviews will be contacted for further information if required. For the full MEDLINE search strategy, see *Extended data* ^38^. 

**Searching other resources**

Handsearching of the journal Implementation Science (2006-2022), BMJ Quality and Safety (2005-2022) and two new journals, Implementation Science Communication (2020, 2021, 2022) and Implementation Research and Practice (2020, 2021, 2022) will also be completed using keywords and Boolean operators. Grey literature will be searched using Google Scholar, using the title-only function, and screening the first 1000 records in accordance with guidance from Haddaway *et al*. ^39^, Seminal reviews ^4^, commentaries ^3^ and articles (The Tailored Implementation in Chronic Diseases (TICD) study ^9, 40^ and ImpleMentAll) will be included and their reference lists searched. Lastly, implementation process frameworks (e.g., KTA, Exploration Preparation Implementation Sustainment – EPIS - framework) will be identified and included to understand how tailoring has been conceptualised and inform Objective 1.

Articles retrieved from each database will be imported as .ris files into Covidence where duplicates will be removed. Covidence has been shown to overperform reference managers in de-duplication ^41^.

***Stage 3: study selection***

The screening process will be carried out using Covidence. Titles/abstract and full text screening will be split between two or more reviewers. The title/abstract screening process will be piloted initially – 250 titles and abstracts will be screened by the review team using the predefined eligibility criteria. If any discrepancies are identified, these will be discussed within the team. Inter-rater reliability will be calculated for title and abstract screening using the kappa ( *κ*) statistic to indicate alignment during the piloting stage. The screening process will involve double screening. Two reviewers will screen each title and abstract, with conflicts resolved by consensus or a third reviewer. Full texts will also be screened in duplicate with conflicts resolved by consensus or a third reviewer. Reasons for exclusion of full-text articles will be documented to PCC in Covidence. The final search results will be outlined in a PRISMA flow diagram from the PRISMA-ScR statement.

We anticipate that there may be large projects which result in multiple, related but separate publications. For example, authors may report the tailoring process in one publication but an evaluation of the tailored strategy in another, or they may present identification and prioritisation in one publication but strategy selection in a separate publication.

Overall, we will aim to be inclusive when screening title and abstracts, for example, we will include cost-effectiveness studies and evaluations of tailored strategies. We will exclude these studies at the full text screening stage if they do not detail the tailoring process or refer to related publications which describe the process. We will check these papers for references to related studies. If there are multiple papers relating to the same study, not all will be included; we will only include papers from a study if they provide information on the tailoring process and the tailored intervention.

***Stage 4: charting the data***

Data will be extracted by at least two members of the research team using Covidence or into Microsoft Excel software following guidelines from JBI ^26^. Each member will extract data for a proportion of articles. The data extraction form will be pilot tested by the reviewers on 10% of full text articles to ensure that data is consistently extracted. After pilot testing, any discrepancies that arise will be discussed with the rest of the team, and any refinements to the form will be made, if required.

Data to be extracted will include study characteristics and characteristics of tailoring relating to our research questions. Arksey and O’Malley suggest charting the data according to research themes ^25^. The information to be extracted ( Table 2) aligns with the review objectives and is intended to capture key findings that relate to the scoping review question(s).

**Table 2.** Data charting elements.

Publication details	Associated question(s)	Research question
Study title		
Author(s)		-
Year of publication	What year was the study/document published?	-
Publication type	Is the document a peer-reviewed, published article or grey literature?	-
Origin/country of origin	Which country is the study/document focusing on?	-
**General details**		
Aims/purpose	What are the study/document aims?	-
Study design	What is the study/document design?	-
Study setting/healthcare context	What is the described healthcare setting? (Where this information is available, describe the setting in terms of local/team level, organisational level, and external health system level)	9
Study population	Which is the target population of the tailoring approach?	-
**Content**		
Details of the evidence-based intervention whose implementation is being tailored	What intervention is being implemented? What is the aim of the intervention? What is the health-related focus of the study/document (health condition focused on)? What is the study population?	- - -
Details of the tailoring approach	Is tailoring used prospectively to develop implementation strategy?	8
	Is there a rationale for tailoring? What is the rationale? Is tailoring based on a theory? What is the theory?	2 2 3 3
	Was a theory or framework drawn on during tailoring process? Which framework/theory? At what stage?	7 7 7
	What steps are involved?	6
	When is tailoring carried out (stage of implementation)?	8
	Who is involved: health care professionals, researchers and/or people who used healthcare services? When (at what stage(s)) are they involved), for what purpose (what is their role), and how are they involved?	10 10
	Has determinant prioritisation been conducted?	11
	Has strategy selection been conducted?	11
	How has the determinant prioritisation and strategy selection step of tailoring been conducted? What criteria have been used in prioritisation? Who selected these criteria? What data collection or broader methods have been used to inform tailoring? Is there a rationale for the methods used for this step? What is the rationale? What are the inputs used to inform strategy selection – for example, evidence, stakeholders, theory? What is the balance and timing of each input?	11 11 11 11a 11a 11a 11b 11c
Outcome(s) of tailoring process and how measured.	Was tailoring evaluated? How was tailoring evaluated? What proximal outcomes of tailoring “success” were used?	12 12 12

**Assessment of methodological quality**

Critical appraisal of studies included in scoping reviews is not consistently performed but is increasingly recommended to facilitate the interpretation of the results and support their uptake into to policy and practice ^24^. However, as the aim of this review is to describe and understand how tailoring has been conducted, undertaking a quality assessment of included studies is less relevant. The tailoring literature is voluminous and, as indicated by the different definitions, is potentially heterogenous, therefore we considered quality assessment beyond the review scope. A tailoring process may involve several studies and different methods (i.e., surveys, focus groups, interviews) and so it would be challenging to provide an overall assessment of a tailoring process, and such assessment would be unlikely to inform review conclusions regarding the evidence gaps and future research. Lastly, as flagged by Levac *et al*. ^24^, it is unclear how grey literature may be evaluated (Stage 6).

***Stage 5: collating, summarising, and reporting the results***

Levac *et al.,* ^24^ advise scoping review authors to divide Stage 5 into three distinct steps: analysing the data (collating and summarising), reporting results, and applying meaning to the results.

*Step 1: collating and summarising the results (analysis)*

Initial analysis will be quantitative involving a descriptive numerical summary of the characteristics of the studies (e.g., number of studies, publication years, study populations) and of the tailoring process (e.g., types and number of stakeholders involved, timing of tailoring). Deductive content analysis aligned to the research questions will be undertaken, to prepare a narrative summary to accompany the tabular results. Inductive thematic analysis, following the guidance of Braun and Clarke ^42^, will be conducted to develop themes related to evidence gaps. If there is insufficient data for inductive thematic analysis, deductive analysis will be used to capture the descriptive information which is available (or not) e.g., items can be categorised as ‘missing’. NVivo will used to manage qualitative analysis where applicable. Evidence gaps will broadly be organised according to the study objectives (i.e., conceptualisation, operationalisation, evaluation). For example, under the research question ‘How was the prioritisation of determinants and selection of strategies conducted?’ a theme could be ‘variable criteria used for prioritisation’. We would propose the gaps are potentially (1) lack of evidence on which criteria to use to prioritise determinants and (2) lack of evidence on the impact of the criteria chosen. Proposed evidence gaps will be reviewed as part of the stakeholder consultation (Stage 6). Where appropriate we will develop visual depictions (e.g., geographic or topical visualisation) to support thematic summaries of the evidence gaps.

*Step 2: reporting*

Results will be reported using the PRISMA-ScR guidelines. A PRISMA flow diagram will be presented to show the study selection and reasons for exclusion at full text review. Quantitative results will be presented in tabular form (Objective 2), and as a narrative summary organised according to the review questions: tailoring definitions and conceptualisation (Objective 1), operationalisation (Objective 2), evaluation (Objective 3) and evidence gaps (Objective 4). Evidence gaps will be identified by considering what information is missing in the description of tailoring, whether there are research questions which cannot be answered or only partly answered based on the available literature, where the most variation exists across different studies and where there is uncertainty about the way to approach an aspect of tailoring. We will record and summarise where there is insufficient data on aspects of tailoring to inform our findings on the tailoring evidence gap

The results will be reported with a view to achieving the following goals:

- Improved understanding of how tailoring has been defined, and carried out (i.e., what is commonly done and why?)
- Enhanced understanding of what information is used to guide determinant or strategy prioritisation
- Creation of a ‘menu’ of tailoring approaches used to date.
- Enhanced clarity on the core ingredients of tailoring and the balance of these elements.
- Comprehensive overview of gaps in the evidence.

*Step 3: Implications*

The review findings will help develop an understanding of how tailoring is conceptualised, operationalised, and evaluated within the literature. The broader implications of the findings for policy, practice and research will be highlighted.

***Stage 6: Consultation with stakeholders***

The purpose of the stakeholder consultation is to advise on the content and priorities of a tailoring research agenda and on the dissemination of review findings. This review is part of a larger research project on the tailoring process in healthcare. The larger project involves an international scientific advisory panel of experts in the field of implementation science working in different academic and health service entities. This group will be consulted, along with project stakeholders who represent health service organisations, during later phases of the analysis and interpretation of the results (Stage 5). The preliminary format of consultation will be through a structured discussion/focus group. Stakeholders will be presented with the proposed evidence gaps, organised by theme; for example, ‘decision-making during determinant prioritisation’. Agreement with the gap and its categorisation will be checked. Then, based on the agreed gaps, stakeholders will be asked to identify priorities for a research agenda focused on tailored approaches to implementation. The consultation will also inform how to present the findings in a format useful for implementation researchers and practitioners, and how best to disseminate the findings. The consultation process represents a knowledge exchange opportunity, and stakeholders will be able to build on the findings, bring different perspectives, and identify strategies for disseminating review findings. This will help ensure the findings have practical relevance. Further consultation will be considered based on recommendations from the scientific advisory panel. If we proceed with further consultation, an invitation will be issued to stakeholders via co-author networks and through relevant professional societies and organisations.

## Discussion

Careful use of strategies tailored to address important barriers to, and enablers of, implementation is essential to support the successful adoption and delivery of EBIs. Despite tailoring becoming more common, we lack clarity on definitions, methods, and principles of tailoring. Currently no synthesis of the existing evidence on the tailoring process exists. While also systematic, scoping reviews are distinguished from systematic reviews by having broader research question(s), with the aim of identifying the scope of literature on a subject, gaps in the evidence, rather than that of formally assessing the quality of evidence and generating a conclusion in response to a focused research question ^43^. This scoping review will describe how tailoring has been undertaken within the healthcare context, identify research gaps, and inform priorities for a research programme on tailoring. This review can be used as a resource by the implementation science community and healthcare practitioners involved in implementation, to guide future research in this field and facilitate systematic, transparent, and replicable development of tailored implementation strategies.

### Study status

The initial searches are currently underway at the time of this article publication, and the MEDLINE search strategy has been developed. We intend to disseminate the results through publication in a peer-reviewed journals, conference presentations, and research summaries.

## Data Availability

All data underlying the results are available as part of the article. Open Science Framework: Characterising processes and outcomes of tailoring implementation strategies in healthcare: A scoping review https://doi.org/10.17605/OSF.IO/ER9HY ^38^ This project contains the following extended data: MEDLINE search strategy.pdf (MEDLINE search strategy) Eligibility criteria.pdf (Study eligibility criteria) Data charting elements.pdf (Elements to be extracted from included studies) PRISMA-P 2015 checklist.pdf Data are available under the terms of the Creative Commons Attribution 4.0 International license (CC-BY 4.0).

## Software availability

Covidence is a proprietary software. An alternative software which can be used for review management is Rayyan or EPPI reviewer.
