# Supplementary material for: Characterising processes and outcomes of tailoring implementation strategies in healthcare: a protocol for a scoping review
Source: HRB Open Res. 2022 Sep 20;5:17. Originally published 2022 Mar 3. [Version 2] doi: 10.12688/hrbopenres.13507.2 (PMC10853668; doi:10.12688/hrbopenres.13507.2)
Supplement: Peer review history

# Reviewer report: Reviewer response for version 2

**DOI:** 10.21956/hrbopenres.14882.r37269

**Published:** 2024-02-08

**Authors:** Thomas von Lengerke

**Affiliations:** 1 Department of Medical Psychology, Center for Public Health and Health Care, Hannover Medical School, Hannover, Germany

This article presents a protocol for a scoping review on processes and outcomes of tailoring implementation strategies in healthcare. In a nutshell, it aims to explore how tailoring had been defined/conceptualised, operationalized, and evaluated in healthcare so far. With the framework and principles of (Arksey and O'Malley , 2005) [Ref 1] and (the refinements of Levac et al, 2010) [Ref 2]., the JBI guidance(Micah DJ Peters et al,2020) [Ref 3], and the PRISMA-ScR guidelines (Tricco AC et al,2018) [Ref 4], in my view all relevant scoping review recommendations have been appropriately considered.

 As the article has already been subject to one round of reviewing and revision, many issues have already been, in my view, sufficiently dealt with, among others exclusion of clinical interventions in precision medicine tailored to patient characteristics, how to deal with multiple related, but separate publications from individual projects, and the role of grey literature. Thus, I can only add a limited number of suggestions:

 a.) Fourth aim (see "Purpose of conducting the scoping review"): Isn't the identification of knowledge gaps and future research priorities always one aim of (scoping) reviews? That is, doesn't this aim go without saying?

 b.) Broad definition of tailoring (see "Purpose of conducting the scoping review"): Ultimately, tailoring refers to actions/activities/behaviours of proxy agents and implementation support practitioners. If this is what is meant with "proactive process" and "modifications of an" in the definition, that's fine; yet, I suggest to think about being more explicit here, e.g. by changing the second wording into "proactively modifying an".

 c.) Please add information on how the scoping review will deal with implementation interventions tailored to proxy agents/implementation support practitioners characteristics (second-order implementation

 interventions [1]) in contrast to implementation interventions tailored to clinicians (first-order implementation interventions [1]). For example, one can tailor implementation interventions to support clinicians hand hygiene (first-order), or one can tailor implementation interventions such as train-the-trainer workshops.to improve the quality of educational trainings on hand hygiene (which are eventually provided by infection and control teams to clinicians - second-order). Will the review include second-order implementations interventions as well (which would be of added value since they ultimately aim to enhance the adoption, implementation, and sustainment of clinical programs or practices as well)? And if so. How will the two different interventions types be handled and presented?

Is the study design appropriate for the research question?

Yes

Is the rationale for, and objectives of, the study clearly described?

Yes

Are sufficient details of the methods provided to allow replication by others?

Yes

Are the datasets clearly presented in a useable and accessible format?

Not applicable

Reviewer Expertise:

preventing healthcare-associated infections by promoting professional compliance with evidence-based guidelines; population hand hygiene behaviour; public health psychology

I confirm that I have read this submission and believe that I have an appropriate level of expertise to confirm that it is of an acceptable scientific standard.

## References

[1] : Scoping studies: towards a methodological framework. *International journal of social research methodology* .2005;8(1) : 10.1080/1364557032000119616 10.1080/1364557032000119616

[2] : Scoping studies: advancing the methodology. *Implement Sci* .2010;5: 10.1186/1748-5908-5-69 69 10.1186/1748-5908-5-69 20854677 PMC2954944

[3] : JBI Manual for Evidence Synthesis. *JBI/year* . 10.46658/JBIMES-20-12 10.46658/JBIMES-20-12

[4] : PRISMA Extension for Scoping Reviews (PRISMA-ScR): Checklist and Explanation. *Ann Intern Med* .2018;169(7) : 10.7326/M18-0850 467-473 10.7326/M18-0850 30178033

# Reviewer report: Reviewer response for version 2

**DOI:** 10.21956/hrbopenres.14882.r32881

**Published:** 2022-11-15

**Authors:** Craig S. Rosen

**Affiliations:** 1 Dissemination & Training Division, National Center for PTSD, VA Palo Alto Health Care System, Menlo Park, CA, USA

The revisions are all appropriate and strengthen the protocol. I would propose accepting as is.

Is the study design appropriate for the research question?

Yes

Is the rationale for, and objectives of, the study clearly described?

Yes

Are sufficient details of the methods provided to allow replication by others?

Yes

Are the datasets clearly presented in a useable and accessible format?

Not applicable

Reviewer Expertise:

Implementation science; mental health services reseach; posttraumatic stress

I confirm that I have read this submission and believe that I have an appropriate level of expertise to confirm that it is of an acceptable scientific standard.

# Reviewer report: Reviewer response for version 1

**DOI:** 10.21956/hrbopenres.14736.r31980

**Published:** 2022-06-07

**Authors:** Craig S. Rosen

**Affiliations:** 1 Dissemination & Training Division, National Center for PTSD, VA Palo Alto Health Care System, Menlo Park, CA, USA

This submission outlines a protocol for a scoping review of tailoring of implementation strategies in health care. The rationale and review of prior literature are clear and compelling. The methods are clearly described and appear appropriate.  Some small modifications might strengthen it further.

1. The first sentence under 'purpose of conducting the scoping review':  "Despite varying definitions and lack of clarity... tailoring is recommended.." seems backwards to me. It might be clearer to say that tailoring is commonly recommended yet there is a lack of clarity on what tailoring is and how it should be done.   
2. p.4 Inclusion criteria and conceptualization. It would help to be explicit here that the review of how has tailoring been defined and conceptualized in healthcare contexts will be limited to the tailoring of “methods or techniques used to enhance the adoption, implementation, and sustainment of a clinical program or practice”. Without this clarification, “tailoring in health care contexts” might include precision medicine tailoring of clinical interventions to patient characteristics.
3. p.5. The listed “justification” for Population in Table 1 does not seem to fit and seems redundant with the justification for Context.  An alternative justification might be that all populations involved in health care research are relevant.
4. Table 2 – the listed study question for ‘authors’ appears incorrect.

Is the study design appropriate for the research question?

Yes

Is the rationale for, and objectives of, the study clearly described?

Yes

Are sufficient details of the methods provided to allow replication by others?

Yes

Are the datasets clearly presented in a useable and accessible format?

Not applicable

Reviewer Expertise:

Implementation science; mental health services reseach; posttraumatic stress

I confirm that I have read this submission and believe that I have an appropriate level of expertise to confirm that it is of an acceptable scientific standard, however I have significant reservations, as outlined above.

# Response

**Published:** 2022-09-19

**Authors:** Fiona Riordan

**Affiliations:** University College Cork, Ireland

Thank you for reviewing our paper and for the suggested amendments.

**The first sentence under 'purpose of conducting the scoping review':  "Despite varying definitions and lack of clarity... tailoring is recommended.." seems backwards to me. It might be clearer to say that tailoring is commonly recommended yet there is a lack of clarity on what tailoring is and how it should be done.**

 We have amended the text (pg 6):

*Despite varying definitions and an apparent lack of clarity about how to best conduct it in practice, tailoring is commonly recommended to support the successful delivery of EBIs.*

**p.4 Inclusion criteria and conceptualization. It would help to be explicit here that the review of how has tailoring been defined and conceptualized in healthcare contexts will be limited to the tailoring of “methods or techniques used to enhance the adoption, implementation, and sustainment of a clinical program or practice”. Without this clarification, “tailoring in health care contexts” might include precision medicine tailoring of clinical interventions to patient characteristics.**

 Thank you for this suggested clarification. We have amended the conceptualisation objective (pg 7) and inclusion criteria in Table 1 and pg 10 as below:

***Objective 1: Conceptualisation***

- *1.* *How has tailoring of implementation strategies (i.e. methods or techniques used to enhance the adoption, implementation, and sustainment of a clinical program or practice) been defined and conceptualised in healthcare contexts?*
- *2.* *Is there a rationale for tailoring and what is the rationale?*
- *3.* *Is tailoring based on a theory which explains why it should work?*

***Context***

*Studies which use a tailoring process to develop strategies (methods or techniques used to enhance the adoption, implementation, and sustainment of a clinical program or practice) for application in healthcare settings will be included.*

**p.5. The listed “justification” for Population in Table 1 does not seem to fit and seems redundant with the justification for Context.  An alternative justification might be that all populations involved in health care research are relevant.**

 We recognise the reviewers point and have amended this on pg. 8 and in Table 1.

 Table 1:

*All* *populations involved in health care research are relevant.* *This could include healthcare professionals, implementation researchers, and* *other people who plan, provide and/or use health services*

# Reviewer report: Reviewer response for version 1

**DOI:** 10.21956/hrbopenres.14736.r31612

**Published:** 2022-03-23

**Authors:** Elaine Yuen Ling Kwok

**Affiliations:** 1 CanChild, Institute of Applied Health Sciences, McMaster University, Hamilton, ON, Canada; 2 Northwestern University, Evanston, IL, USA

Thank you for the opportunity to review this study protocol. Overall, I am pleased with the research question and think that an appropriate plan has been developed for the review. I left several comments below which I hope will be helpful to the authors.

 Is the rationale for, and objectives of, the study clearly described?

- Yes, the objective & rationale of the study are clearly described and I agree with the importance of conducting this scoping review.

Is the study design appropriate for the research question (including statistical power analysis, where appropriate)?

- Research objectives/questions: This part is a strength. Clear objectives. Just a minor comment under objective 2: Consider expanding #5 to describe the context in more depth (i.e., beyond the healthcare setting), perhaps literature on implementation context (e.g., Nilsen & Bernhardsson 2019) ^1^ or determinants framework (e.g., TDF, CFIR) can guide with this data extraction on “context”? Also please consider describing the prioritization of determinants in more depth. I would be very interested to know what criteria were used in prioritizing, and who selected those criteria (e.g., were determinants prioritized based on patients’ outcomes/implementation outcomes…)?
- A scoping review methodology is timely and appropriate for the topic. I am raising a few suggestions/ comments for the authors to consider, and hope these comments will help improve the impact of your review:
  - Scope of the literature searched: Appropriate use of search strategies, although some justifications will help strengthen the decision to only include literature starting in year 2005. Another comment is for authors to consider how they may handle large “tailoring” projects that resulted in multiple related, but separate publications e.g., some authors may choose to report their evaluation & prioritization of implementation determinants in a separate article from their “tailoring” phase, and then a separate article from their evaluation of the tailored implementation strategy phase. Just wondering how your search strategy can capture these related studies?
  - Inclusion criteria: Would be helpful to explain why grey literature is excluded in this review. Given the fact that scoping review allows for the inclusion of grey literature (e.g., conference proceedings/ presentation, government/not-for-profit project reports, grant applications/reports), I think it would be a great opportunity to capture how “tailoring” happens in the “real-world” by including non-traditional work (i.e., non-academic literature).
  - Study selection: No major concerns. Just noting the individual responsible for abstract and full-text screening should probably be trained to understand and to be able consistently apply the definition of “tailoring” & inclusion criteria in this project- this may be tough as the concept of “tailoring, as the authors pointed out, is still poorly defined.
  - Charting the data: I really appreciate the mapping of your data extraction plan to your research objectives/questions. This part is clear to me. Please consider my comments under ‘Research objectives/ questions” above.
  - Collating & summarizing results: The plan to summarize and described the studies is clear and I don’t have major concerns. The plan to highlight the gaps in evidence should be expanded. Can you give an example of how you will do that? Will you lean on an existing implementation framework to help identify these gaps? Or what will be the specific methodologies involved in this step?
  - Consultation with stakeholders: It would help to understand how you plan to identify and recruit the stakeholders and what inclusion criteria will you use (e.g., would you include only stakeholders with tailoring experience? Will they represent various healthcare settings?). Also, I would recommend a more focused stakeholder engagement plan, perhaps more specific to this scoping review. As it currently reads, stakeholders are engaged in this phase to, for example, *suggest further resources*, in that case, will these resources suggested by stakeholders be included in the review as well? If so, please add that to the search strategy description. It seems that stakeholders are also engaged to formulate a research agenda and to help identify dissemination plans. So is the outcome of this scoping review to inform a research agenda? If so, please add that to the objectives? Or perhaps I have mistaken the purpose of this step and that stakeholders are only engaged to help identify gaps in the literature by bringing in their on-the-ground tailoring experience?

Are sufficient details of the methods provided to allow replication by others?

- Yes, see comments above.

Have the authors pre-specified sufficient outcome-neutral tests for ensuring that the results obtained can test the stated hypotheses, including positive controls and quality checks?

- No major concerns here. One possible scenario, in my mind, is the amount of missing information the author may identify in the existing “tailoring” literature. Knowing that our field lacks conceptual clarity around “tailoring” or a reporting guideline, I am, of course, not suggesting it is the fault of any authors who already published their “tailoring” studies. I would be very interested to know how the authors of this review plan to address a potentially “patchy” amount of data available to them in answering each of their research questions (e.g., what if there weren’t sufficient data for an inductive analysis for certain question)?

Are the datasets clearly presented in a useable and accessible format?

- N/A.

Is the study design appropriate for the research question?

Partly

Is the rationale for, and objectives of, the study clearly described?

Yes

Are sufficient details of the methods provided to allow replication by others?

Yes

Are the datasets clearly presented in a useable and accessible format?

Not applicable

Reviewer Expertise:

Rehabilitation/Clinical Services Research

I confirm that I have read this submission and believe that I have an appropriate level of expertise to confirm that it is of an acceptable scientific standard, however I have significant reservations, as outlined above.

## References

[1] : Context matters in implementation science: a scoping review of determinant frameworks that describe contextual determinants for implementation outcomes. *BMC Health Serv Res* .2019;19(1) : 10.1186/s12913-019-4015-3 189 10.1186/s12913-019-4015-3 30909897 PMC6432749

# Response

**Published:** 2022-09-22

**Authors:** Fiona Riordan

**Affiliations:** University College Cork, Ireland

**Research objectives/questions: This part is a strength. Clear objectives. Just a minor comment under objective 2: Consider expanding #5 to describe the context in more depth (i.e., beyond the healthcare setting), perhaps literature on implementation context (e.g., Nilsen & Bernhardsson 2019) or determinants framework (e.g., TDF, CFIR) can guide with this data extraction on “context”?**

 Thank you for reviewing our paper and your suggestions, these are greatly appreciated.

 We agree there is value in exploring tailoring in other settings beyond healthcare settings. However, we have limited the scope of review to healthcare settings given the resources and time available. Given the ambiguity in terms of what constitutes tailoring and that the types of studies we may identify for this review are quite broad, we needed to introduce some boundaries on other criteria to make it more manageable.

 Within the healthcare setting, we will examine the different *levels* of setting, that is the local level, wider organizational level, and external health system level. This has been added as part of data extraction.  We have amended the text in Table 2 to capture this:

*What is the described healthcare setting? (Where this information is available, describe the setting in terms of local/team level, organisational level, and external health system level)*

**Also please consider describing the prioritization of determinants in more depth. I would be very interested to know what criteria were used in prioritizing, and who selected those criteria (e.g., were determinants prioritized based on patients’ outcomes/implementation outcomes…)?**

 We will describe the prioritisation of determinants in greater detail. We have amended Table 2 to reflect this:

- *How has the prioritisation and selection step of tailoring been conducted? *
- *What criteria have been used in prioritisation? *
- *Who selected these criteria?*
- *What data collection or broader methods have been used*
- *Is there a rationale for the methods used for this step?*
- *What is the rationale? *
- *What are the inputs during strategy selection – evidence, stakeholders, theory? *
- *What is the balance and timing of each input?*

**A scoping review methodology is timely and appropriate for the topic. I am raising a few suggestions/ comments for the authors to consider, and hope these comments will help improve the impact of your review:**

**Scope of the literature searched: Appropriate use of search strategies, although some justifications will help strengthen the decision to only include literature starting in year 2005.**

 We have expanded on our justification in Table 1:

*Given the breadth of the search and resources available, the decision was made to focus on over 17 years of literature. The flagship journal in the field, Implementation Science started in 2006, signalling the formal establishment of the field in 2006 and subsequently, dedicated journals, including Implementation Science Communications (2020), Implementation Research and Practice (2020), and Global Implementation Research and Applications (2021) have been established.*

 We have also added the following to the main text (’Context’) (pg 11):

A preliminary electronic search for key terms  (“tailor*” and terms for strategy or intervention and implementation and healthcare) in title and abstracts along with relevant subject headings in MEDLINE (14) indicates that in the last 20 years, there has been an upward trend in articles referring to tailoring, with 1722 articles published 2012-2021 compared to 443 articles in the 10 years (2002-2011) previous. In total there were 152 articles published 1972 – 2005 and 2148 from 2006 to 2022. Hence our decision regarding search date limitations.

 To note, we also include additional clarification on why we completed dedicated searches for a specific tailoring approach, ‘implementation mapping’ (pg.11):

*Dedicated searches for a specific tailoring approach, ‘implementation mapping’, will be completed for each database. This specific approach was chosen as it is unique to tailoring implementation strategies. Other methods (intervention mapping concept mapping, conjoint analysis, and group model building) while suggested as possible tailoring approaches, can also be used in other fields and for other purposes. This lack of specificity was confirmed during preliminary searches conducted to inform search development.*

 We also wished to take this opportunity to be more explicit in terms of our eligibility, namely that studies which use ‘implementation mapping’ term will be included as this is taken to be an approach to tailoring (pg. 8):

*While there is variation in the scope of tailoring (i.e., whether determinant identification and/or prioritisation, and strategy selection, and/or application and testing are included), strategy selection is a core aspect of tailoring. Authors must explicitly describe the process as tailoring or the strategy as tailored.  Studies will also be included if they refer to the intervention/strategy development process as ‘implementation mapping’. This term was adapted from ‘intervention mapping’ and first used by Fernandez et al to specifically describe an approach to tailor implementation strategies.*

 We also provided further clarification on dealing with ambiguity between evidence-based interventions and implementation strategies (pg. 11):

*We will commit to the definition of strategy cited above: “methods or techniques used to enhance the adoption, implementation, and sustainment of a clinical program or practice” ^1 ^. For this definition to apply there must be a clearly identifiable intervention (program, practice, guideline, policy or innovation) being implemented. The focus will not be on the nature or type of intervention being implemented, or whether it should be implemented, rather the focus will be on the tailored strategy supporting implementation. Where the delineation between intervention and strategy is unclear, we will refer to the ERIC taxonomy to identify implementation strategies. At the full text screening stage, if we cannot clearly delineate the intervention, we will contact the study authors for more information. If the author cannot provide more clarity, we will include the study but report the ambiguity.*

**Another comment is for authors to consider how they may handle large “tailoring” projects that resulted in multiple related, but separate publications e.g., some authors may choose to report their evaluation & prioritization of implementation determinants in a separate article from their “tailoring” phase, and then a separate article from their evaluation of the tailored implementation strategy phase. Just wondering how your search strategy can capture these related studies?**

 We agree. This is something we have discussed when piloting our screening approach.

 We expect that the search strategy will capture multiple articles from the same study. As suggested, some may have separate articles which cover the evaluation of a tailored intervention or strategy. During title and abstract screening, we will be inclusive. We will include cost-effectiveness studies and evaluations of tailored strategies. During full text screening these ‘results’ papers will be checked for reference to the tailoring phase.  We will exclude them at full text screening stage if they do not provide further details on the tailoring process (in line with our eligibility criteria) or refer to a related publication which describes this process. If there are multiple papers relating to the same study, not all will be included; we will only include papers from a study if they provide information on the tailoring process and the tailored intervention.

 Under our description of study selection (pg. 12), we include some additional details on this point:

*We anticipate that there may be large projects which result in multiple, related but separate publications. For example, authors may report the tailoring process in one publication but an evaluation of the tailored strategy in another, or they may present identification and prioritisation in one publication but strategy selection in a separate publication.*

*Overall, we will aim to be inclusive when screening title and abstracts, for example, we will include cost-effectiveness studies and evaluations of tailored strategies. We will exclude these studies at the full text screening stage if they do not detail the tailoring process or refer to related publications which describe the process. We will check these papers for references to related studies. If there are multiple papers relating to the same study, not all will be included; we will only include papers from a study if they provide information on the tailoring process and the tailored intervention.*

**Inclusion criteria: Would be helpful to explain why grey literature is excluded in this review. Given the fact that scoping review allows for the inclusion of grey literature (e.g., conference proceedings/ presentation, government/not-for-profit project reports, grant applications/reports), I think it would be a great opportunity to capture how “tailoring” happens in the “real-world” by including non-traditional work (i.e., non-academic literature).**

 Grey literature will be searched, albeit in a limited way, using Google Scholar, using the title-only function, and screening the first 1000 records. We will include some unpublished (grey literature) including theses/dissertations, book chapters, toolkits, guidance, and reports but exclude conference abstracts and other non-peer reviewed sources (i.e., presentations, websites, blogs, studies in progress) (pg. 9 and Table 1). We have amended Table 1 to clarify the nature of the grey literature.

 Originally, we limited the grey literature search due to resource constraints and our uncertainty as to whether it would be worth the yield, for example, searching organisational websites to identify reports. We suggest that future work could look more extensively at the application of tailoring in non-academic literature.

 Please note that we have also amended the related question in Table 2 Data charting elements (pg 13):

*Is the document a peer-reviewed, published article or grey literature?*

**Study selection: No major concerns. Just noting the individual responsible for abstract and full-text screening should probably be trained to understand and to be able consistently apply the definition of “tailoring” & inclusion criteria in this project- this may be tough as the concept of “tailoring, as the authors pointed out, is still poorly defined.**

 We have expanded our review team and piloted our screening approach to ensure we consistently apply the definition and inclusion criteria, holding a number of alignment meetings to discuss uncertainties. We limited the pilot to 250 articles, so that we could dedicate more time to discussion and reflect on any discrepancies. We held a number of meetings during the piloting process to discuss the application of eligibility criteria. As it is difficult to apply the concept of tailoring, we have agreed to be inclusive at the title and abstract screening stage, recognising that this will lead to a larger number of full texts to screen. We have also decided to apply double screening.  All reviewers have an opportunity to practice applying the screening questions.

 We have amended the text (pg 12) as follows:

*The screening process will be carried out using Covidence.* *Titles/abstract and full text screening will be split between two or more reviewers. The title/abstract screening process will be piloted initially – 250 titles and abstracts will be screened by the review team using the predefined eligibility criteria. If any discrepancies are identified, these will be discussed within the team. Inter-rater reliability will be calculated for title and abstract screening using the kappa (κ) statistic to indicate alignment during the piloting stage.  The screening process will involve double-screening. Two reviewers will screen each title and abstract, with conflicts resolved by consensus or a third reviewer. Full texts will also be screened in duplicate with conflicts resolved by consensus or a third reviewer. Reasons for exclusion of full-text articles will be documented to PCC in Covidence. The final search results will be outlined in a PRISMA flow diagram from the PRISMA-ScR statement.*

*We anticipate that there may be large projects which result in multiple, related but separate publications. For example, authors may report the tailoring process in one publication but an evaluation of the tailored strategy in another, or they may present identification and prioritisation in one publication but strategy selection in a separate publication.*

*Overall, we will aim to be inclusive when screening title and abstracts, for example, we will include cost-effectiveness studies and evaluations of tailored strategies. We will exclude these studies at the full text screening stage if they do not detail the tailoring process or refer to related publications which describe the process. We will check these papers for references to related studies. If there are multiple papers relating to the same study, not all will be included; we will only include papers from a study if they provide information on the tailoring process and the tailored intervention.*

**Charting the data: I really appreciate the mapping of your data extraction plan to your research objectives/questions. This part is clear to me. Please consider my comments under ‘Research objectives/ questions” above.**

 We have reflected on data extraction in study context above and made amendments to capture different levels within the healthcare setting.

**Collating & summarizing results: The plan to summarize and described the studies is clear and I don’t have major concerns. The plan to highlight the gaps in evidence should be expanded. Can you give an example of how you will do that? Will you lean on an existing implementation framework to help identify these gaps? Or what will be the specific methodologies involved in this step?**

 Our process to identify and highlight evidence gaps will involve:

- Thematic analysis
- Visual depiction (where appropriate)
- Stakeholder consultation

As outlined in the protocol, evidence gaps will be identified by considering the following: what information is missing in the description of tailoring, whether there are research questions which cannot be answered or only partly answered based on the available literature, where the most variation exists across different studies, and where there is uncertainty about the way to approach an aspect of tailoring. They will be organised according to the study objectives (i.e., conceptualisation, operationalisation, evaluation).

 In terms of visualisation, there is little consensus on the exact definition of what constitutes an evidence gap map but broadly speaking it is considered a systematic search to identify gaps in knowledge and/or future research needs, presenting these in a user-friendly format e.g., a visual figure or graph, or a searchable database (Miake-Lye *et al.*, 2016).

 Miake-Lye, I.M., Hempel, S., Shanman, R. *et al.* What is an evidence map? A systematic review of published evidence maps and their definitions, methods, and products. *Syst Rev* **5**, 28 (2016).

 We have added some text to elaborate on the approach in our ‘Analysis’ section (pg 15):

*Deductive content analysis aligned to the research questions will be undertaken, to prepare a narrative summary to accompany the tabular results. Inductive thematic analysis, following the guidance of Braun and Clarke ^41^, will be conducted to develop themes related to evidence gaps. Evidence gaps will broadly be organised according to the study objectives (i.e., conceptualisation, operationalisation, evaluation).* *For example, under the research question ‘How was the prioritisation of determinants and selection of strategies conducted?’ a theme could be ‘variable criteria used for prioritisation’. We would propose the gaps are potentially (1) lack of evidence on which criteria to use to prioritise determinants and (2) lack of evidence on the impact of the criteria chosen. Proposed evidence gaps will be reviewed as part of the stakeholder consultation (Stage 6). Where appropriate we will develop visual depictions (e.g., geographic or topical visualisation) to support thematic summaries of the evidence gaps.*

 We have amended the text under ‘Stage 6: consultation with stakeholders’ (pg 16):

*Stakeholders will be presented with the proposed evidence gaps, organised by theme; for example, ‘decision-making during determinant prioritisation’. Agreement with the gap and its categorisation will be checked. Then, based on the agreed gaps, stakeholders will be asked to identify priorities for a research agenda focused on tailored approaches to implementation.*

**Consultation with stakeholders: It would help to understand how you plan to identify and recruit the stakeholders and what inclusion criteria will you use (e.g., would you include only stakeholders with tailoring experience? Will they represent various healthcare settings?).**

**Also, I would recommend a more focused stakeholder engagement plan, perhaps more specific to this scoping review. As it currently reads, stakeholders are engaged in this phase to, for example, suggest further resources, in that case, will these resources suggested by stakeholders be included in the review as well? If so, please add that to the search strategy description.**

 In the first instance stakeholders will involve co-authors (an international group of experts in the implementation science field) and project stakeholders. They will represent academic and healthcare settings. They may not all have direct experience of tailoring but will have experience in and be familiar with implementation of evidence-based practices (science and practice). Further stakeholders to consult may be suggested by this initial group (pg 16).

*The purpose of the stakeholder consultation is to advise on the content and priorities of a tailoring research agenda and on the dissemination of review findings.* *This review is part of a larger research project on the tailoring process in healthcare. The larger project involves an international scientific advisory panel of experts in the field of implementation science working in different academic and health service entities. This group will be consulted, along with project stakeholders who represent health service organisations, during later phases of the analysis and interpretation of the results (Stage 5). The preliminary format of consultation will be through a structured discussion/focus group. Stakeholders will be presented with the proposed evidence gaps, organised by theme; for example, ‘decision-making during determinant prioritisation’. Agreement with the gap and its categorisation will be checked. Then, based on the agreed gaps, stakeholders will be asked to identify priorities for a research agenda focused on tailored approaches to implementation.* *The consultation will also inform how to present the findings in a format useful for implementation researchers and practitioners, and how best to disseminate the findings*. *The consultation process represents a knowledge exchange opportunity, and stakeholders will be able to build on the findings, bring different perspectives, and identify strategies for disseminating review findings. This will help ensure the findings have practical relevance.*

 We will not ask stakeholders to suggest additional resources as it is potentially extending or re-opening the review and given the search is systematic, we expect these resources should be picked up.

**It seems that stakeholders are also engaged to formulate a research agenda and to help identify dissemination plans. So is the outcome of this scoping review to inform a research agenda? If so, please add that to the objectives? Or perhaps I have mistaken the purpose of this step and that stakeholders are only engaged to help identify gaps in the literature by bringing in their on-the-ground tailoring experience?**

 The purpose of the stakeholder consultation is to inform a research agenda for the CUSTOMISE project and advise on dissemination plans for the scoping review results. We have made this more explicit in the objectives (pg 6) and the stakeholder engagement section (pg 16).

*Through seeking to answer the above questions we will determine gaps in the literature (Objective 4) which need to be addressed as part of future research on tailoring and inform a research agenda focused on tailored approaches to implementation (Objective 5).*

**One possible scenario, in my mind, is the amount of missing information the author may identify in the existing “tailoring” literature. Knowing that our field lacks conceptual clarity around “tailoring” or a reporting guideline, I am, of course, not suggesting it is the fault of any authors who already published their “tailoring” studies. I would be very interested to know how the authors of this review plan to address a potentially “patchy” amount of data available to them in answering each of their research questions (e.g., what if there weren’t sufficient data for an inductive analysis for certain question)?**

 We will capture and synthesize the level and completeness of information available on various aspects of tailoring as part of the results of this review. Studies will be included even if they provide minimal data as described below (pg 10):

*Studies will only be included if they (a) describe the tailoring approach in some detail, that is, the authors describe at least one aspect of the tailoring approach (i.e., when it was conducted, who was involved, format/method used, steps involved, inputs) and (b) at least involve the selection of strategies.*

 In cases where data are lacking, we will report this in the results section and use this to inform our findings on research gaps. For example, our findings may point towards the need for systematic reporting of certain aspects of tailoring process.  If there is insufficient data for inductive thematic analysis, the deductive analysis will be used to capture the descriptive information which is available (or not) e.g., items can be categorised as ‘missing’. We have amended text to reflect how minimal data will be analysed (pg. 15) will reported (pg 16):

 Pg. 15:

*Initial analysis will be quantitative involving a descriptive numerical summary of the characteristics of the studies (e.g., number of studies, publication years, study populations) and of the tailoring process (e.g., types and number of stakeholders involved, timing of tailoring). Deductive content analysis aligned to the research questions will be undertaken, to prepare a narrative summary to accompany the tabular results. Inductive thematic analysis, following the guidance of Braun and Clarke ^41 ^, will be conducted to develop themes related to evidence gaps. If there is insufficient data for inductive thematic analysis, deductive analysis will be used to capture the descriptive information which is available (or not) e.g., items can be categorised as ‘missing’.*

 Pg. 16:

*Results will be reported using the PRISMA-ScR guidelines. A PRISMA flow diagram will be presented to show the study selection and reasons for exclusion at full text review. Quantitative results will be presented in tabular form (Objective 2), and as a narrative summary organised according to the review questions: tailoring definitions and conceptualisation (Objective 1), operationalisation (Objective 2), evaluation (Objective 3) and evidence gaps (Objective 4). Evidence gaps will be identified by considering the following: what information is missing in the description of tailoring, whether there are research questions which cannot be answered or only partly answered based on the available literature, where the most variation exists across different studies and where there is uncertainty about the way to approach an aspect of tailoring. We will record and summarise where there is insufficient data on aspects of tailoring to inform our findings on the tailoring evidence gap*
